# Task-specific facilitation of cognition by cathodal transcranial direct current stimulation of the cerebellum

**DOI:** 10.1016/j.brs.2012.03.006

**Published:** 2012-04

**Authors:** Paul A. Pope, R. Chris Miall

**Affiliations:** Behavioural Brain Sciences, School of Psychology, University of Birmingham, Edgbaston, Birmingham B15 2TT, UK

**Keywords:** Cerebellum, tDCS, Cognition

## Abstract

A role for the cerebellum in cognition is controversial, but it is a view that is becoming increasingly popular. The aim of the current study was to investigate this issue using transcranial Direct Current Stimulation (tDCS) during two cognitive tasks that require comparable motor skills, but different levels of working memory and attention. Three groups of twenty-two participants each performed the Paced Auditory Serial Addition Task (PASAT) and a novel variant of this task called the Paced Auditory Serial Subtraction Task (PASST), together with a verb generation task and its two controls, before and after the modulation of cortico*-*cerebellar connectivity using anodal or cathodal tDCS over the cerebellum. Participants’ performance in the difficult PASST task significantly improved after cathodal stimulation compared to sham or anodal stimulation. Improvement in the easier PASAT was equal across all three stimulation conditions. Improvement in verbal response latencies were also greatest during the PASST task after cathodal stimulation, compared to sham and anodal stimulation, and became less variable. Results for the verb generation task complimented those for the PASST, such that the rate and consistency of participants’ verbal responses were facilitated by cathodal stimulation, compared to sham and anodal stimulation. These findings suggest that DC stimulation over the right cerebellum affects working memory and attention differently depending on task difficulty. They support a role for the cerebellum in cognitive aspects of behaviour, whereby activity in the prefrontal cortex is likely dis-inhibited by cathodal tDCS stimulation over the right cerebellar cortex, which normally exerts an overall inhibitory tone on the cerebral cortex. We speculate that the cerebellum is capable of releasing cognitive resources by dis-inhibition of prefrontal regions of cerebral cortex, enhancing performance when tasks become demanding.

## Introduction

For over 200 years the cerebellum has been viewed as an important motor control structure [16,21,26,46] playing a significant role in the prediction, timing and execution of movements [40]. As expected, more is therefore known about the role of this structure in the control of movement than about its role in higher cognitive functions. However, a role for the cerebellum in cognition is suggested by anatomical studies of cerebellar circuits and their connections with the prefrontal cortex [27,30,35,36], by clinical observations of cognitive deficits in patients with local cerebellar lesions [47] and by data from many functional imaging studies, including tests of working memory and language processing [1,15,32,39,42].

Anatomical findings suggest the existence of reciprocal projections from the cerebellum to separate ’motor’ regions (primary motor cortex [M1] and supplementary motor area [SMA]) and ’non-motor’ (the dorsolateral prefrontal cortex [DLPC] and pre-SMA) regions of the cerebrum (reviewed in Ref. [56]). Connectivity between non-motor regions of the cerebellum and the prefrontal cortex is greatly increased in humans relative to primates, and might support higher cognitive functions of the cerebellum [2]. Nonetheless, non-motor connections of the cerebellum with the prefrontal cortex are much less prominent than those connections with skeletomotor, visuomotor and posterior parietal areas of cortex [21].

Evidence for separate motor and non-motor loops between the cerebellum and the cerebrum also helps to explain clinical observations that damage to anterior portions of the cerebellum produce movements marked by a lack of coordination (’dysmetria of movement’), causing ataxia, while damage to posterior portions of the cerebellum is marked by a lack of coordination of intellect and emotion (’dysmetria of thought’), causing the Cerebellar Cognitive Affective Syndrome (CCAS), which affects executive function, spatial cognition, language and personality [48,50,52].

In addition to those anatomical findings and clinic observations, work using brain imaging techniques further support a role for the cerebellum in certain cognitive functions. Using magnetic resonance imaging (MRI), Schmahmann and colleagues have observed regional differences in cerebellar activation patterns depending on specific task demands, also suggesting that the cerebellum can be divided into motor and non-motor regions, based on distinct patterns of functional connectivity between the cerebellum and the cerebrum. In a meta-analysis (based on a limited number of available studies), they identified findings that show cerebellar activity in the normal population for tasks with separate motor, somatosensory, language, verbal working memory, spatial, executive function and emotional processing components [54,55].

In fact, very many brain imaging studies have shown cerebellar activity for a whole range of tasks, which involve all kinds of cognitive operations. While some of these studies have attempted to explain why the cerebellum is engaged, others have either failed to replicate previous findings, or to mention why/how the cerebellum could be involved in cognitive versus motoric task components. So a role for the cerebellum in cognition is still controversial, but the accumulating evidence is beginning to alter conventional wisdom. However, to demonstrate a cerebellar contribution to cognitive tasks, one needs to design experiments that carefully partition out motor and non-motor task components. The present study attempted this by combining, first, a parametric method to vary the level of cognitive relative to motor demands required to perform two separate information processing tasks, and second, a brain stimulation procedure to modulate cerebellar function.

Recent attempts to investigate cerebellar functions have involved a novel form of non-invasive neurostimulation known as transcranial direct current stimulation (tDCS). The method involves delivering low direct current (DC) through a pair of electrodes: one stimulation electrode is placed over the region of interest, and the other reference electrode is placed over the head or shoulder on the opposite side of the body. Intracerebral current flow between the two electrodes excites neurons in the region of interest, producing both neurophysiological and behavioural changes in the participant. It is a potentially valuable alternative approach to studies with cerebellar patients, since the procedure has the capacity to modulate its function. A few studies have been published that demonstrate effects of tDCS on the cerebellum. In one study, polarity-specific effects of DC stimulation were observed on connections between the cerebellum and the prefrontal cortex as tested with a conditioning pulse of transcranial magnetic stimulation (TMS) over different brain regions [19]. In another, cerebellar functions related to adapting fast reaching movements during a visuomotor transformation task were enhanced after the application of tDCS over the cerebellum [20]. With regards to non-motor tasks, only one study involving tDCS over the cerebellum has been published [13], and revealed a modulatory effect of this procedure on verbal working memory.

The Paced Auditory Serial Addition Task (PASAT; [22]) is a neuropsychological test used to assess arithmetic aspects of working memory and attention. It typically involves subjects listening to a series of numbers presented at either 2 or 3 s intervals, and they are required to add the number they hear to the number immediately before it, and then vocalize the answer. This task is difficult as it imposes a high cognitive load, but it is achievable after a short practice block. There are normative data for its performance at different presentation rates [11]. Changing the instructions so that subjects are required to subtract rather than add the two numbers makes the task considerably more difficult, a task we call the Paced Auditory Serial Subtraction Task (PASST). In the general population, learning to perform subtraction is generally more difficult than learning to perform addition, as subtracting one number from another has two order-specific interpretations to consider, unlike adding two numbers together [18]. In this study, our reasoning behind including the two task versions was to make one task considerably more difficult to perform than the other, while keeping motor aspects of the tasks similar. The PASAT and the PASST share the same covert speech operations (comparable motor demands), but require different levels of cognitive skill. If the cerebellum is involved in cognition, one might expect performance to differ between the pre-post-tDCS stimulation sessions, more so during subtraction than during addition.

While no data currently exists for the PASST, brain regions activated by the PASAT have been mapped using positron emission tomography (PET), and include the superior temporal gyrus, the anterior cingulate and bilateral cerebellar sites. These sites are consistent with elements of the task that include auditory perception and processing, speech production, working memory and attention [33]. In an MR scanner, performing the PASAT relative to a control task where subjects merely repeated numbers, involves activity in portions of cerebellar lobule VII for cognitive aspects, but not for motor aspects of the task [24].

We also tested performance on the verb generation task, since generating verbs in response to nouns is an aspect of cognition where the cerebellum has been implicated (see Refs. [15,42]). The Verb Generation Task (VGT; [38,39]) has also been used extensively to investigate a role for the cerebellum during speech/language aspects of working memory and attention. In the VGT, subjects are required to generate verbs in response to nouns, and performance is contrasted with the reading of nouns as a control condition. Both verb generation and noun reading tasks are thought to have similar perceptual and motor demands, but differ in the degree of semantic analysis required to generate a verb versus read a noun. Verb generation requires lexical search processes and verbal response selection, while noun reading requires just reading or naming of single, often over-learnt, items. In the scanner, cerebellar activity is observed when subjects are required to generate a verb versus read a noun, with greater activation of the cerebrocerebellar system, including: left inferior prefrontal cortex, anterior cingulate gyrus and right inferior lateral cerebellum [1,38,39]. Furthermore, when the tasks are repeated across blocks, functional activation changes in the brain systems that support performance. For example, verbs are generated more quickly, and the left prefrontal, cingulate and cerebellar activations are reduced, as seen in both PET [42] and fMRI [53]. However, contrary to these early findings, more recent studies have failed to identify differences between patients and controls on tests of verbal working memory [45], despite the right cerebellar hemisphere being active in healthy subjects performing the same task in the scanner [17].

In this study, cerebellar contributions to arithmetic and language aspects of working memory and attention were assessed in two separate behavioural experiments. In experiment one, performance during the production of two paced arithmetic tasks (addition versus subtraction) was compared before and after the modulation of cortico-cerebellar connectivity using different types of DC stimulation. We hypothesized that, given a role for the cerebellum in cognition, performance during the more cognitively demanding subtraction task would be affected more by DC stimulation than performance on the less demanding task. In experiment two, performance during three language tasks of varying difficulty (verb generation versus noun reading and verb reading) was compared before and after cerebellar stimulation. Unlike some other studies, the words used in this experiment were all related to performing active, rather than passive movements. These language tasks were rated less difficult to perform than the two paced arithmetic tasks. Here, we hypothesized that there might be a weaker effect of cerebellar stimulation in verb generation, relative to the two reading tasks.

## General methods

The two experiments were run in a pseudo-random order across participants, immediately before and after 20 min of DC stimulation. To minimize distractions and allow accurate voice recording, the cognitive tasks were performed inside a quiet cubicle while participants wore a set of Beyerdynamic headphones with a unidirectional microphone (DT234 Pro), which was gated by the amplitude of subjects’ verbal responses and used to measure voice onset times. The presentation of stimuli and the recording of responses was controlled using the Presentation^®^ software (Version 14.2, www.neurobs.com) running on a laptop computer. At the end of both experiments, participants were debriefed about the nature of the experiment. A subjective rating of task difficulty was also obtained from a subset of fifteen participants. They were each asked to rate how difficult each task was to perform on a scale of 1 (easy)–10 (difficult).

### Participants

Sixty six right-handed students at the University of Birmingham with normal vision participated for credit towards a psychology course requirement or for pay, and were arbitrarily allocated into three groups of equal size, receiving anodal (*n* = 22, six male, mean age: 21 yrs), cathodal (*n* = 22, two male, mean age: 20 yrs) or sham (*n* = 22, four male, mean age: 21 yrs) stimulation. All participants gave informed consent and the investigation was approved by the University of Birmingham Ethics Committee.

### Transcranial Direct Current Stimulation

The tDCS over the right cerebellar hemisphere was applied through two sponge electrodes (surface area = 25 cm^2^) moistened with a saline solution. One electrode was centred on the right cerebellar cortex, 1 cm under, and 4 cm lateral to the inion (approximately comparable to the projection of cerebellar lobule VII onto the scalp). The other electrode was positioned on the right deltoid muscle (as in Ref. [13]). The onset and offset of all interventions (anodal, cathodal, and sham) involved current being increased and decreased, respectively, in a ramp-like manner over 10 s (e.g. Refs. [28,37]). The intensity of stimulation was set at 2 mA and delivered over the cerebellum for 20 min using a Magstim DC Stimulator Plus, which is similar to [13]; and considered a safe level of exposure (Iyeretal, 2005), well below the threshold for causing tissue damage [3]. In the sham condition, pseudo stimulation (110uA over 15 ms every 550 ms) was applied for 20 min instead of the stimulation current.

## Experiment one: working memory for paced arithmetic processing

Previous imaging work [24] has demonstrated activation of the lateral cerebellum during paced addition calculations performed in series (PASAT), a demanding cognitive task that involves working memory, attention and arithmetic capabilities. To detect any cerebellar contribution to these processes, we chose to contrast two versions of this task. Thus, three groups of participants performed the paced auditory serial addition task (PASAT) and a novel variant of this task that we called the paced auditory serial subtraction task (PASST). The only difference between the two tasks was the calculation required (addition versus subtraction). To avoid ceiling effects, participants performed each task at an individual difficulty level determined during a preceding practice session. The groups received either anodal (group one), cathodal (group two) or sham (group three) stimulation over the right cerebellum for 20 min.

### Materials

Participants performed a computer version of the traditional PASAT [22] with a modified practice session. The 60 items each contained in the 3s and 2s versions of the PASAT-Form A were used for the addition and subtraction task versions, respectively, *before* the application of tDCS, while the 60 items each contained in the 3s and 2s versions of the PASAT-Form B were used for the addition and subtraction task versions, respectively, *after* tDCS. We included 45 items in the practice sessions as opposed to the 10 practice items in the traditional version. The extra items allowed more time to assess the pace at which subjects could perform each task within a certain limit to avoid a test ceiling effect. The items in each task were different, and the order in which participants performed the PASAT and the PASST was counterbalanced to ensure that performance on one task was not influenced by performance on the other.

### Procedure

Firstly, participants practised the PASAT and the PASST to determine the rate at which auditory items could be presented during the experiment without them making too many errors. This was achieved by increasing the presentation rate of the practice items (reducing the inter-stimulus interval by 300 ms) after every block of five items, between the interval range of 4.2–1.8 s. The point at which participants made 3 errors in a row was noted, and the presentation rate of items preceding this cut-off point was then used in the experimental tasks before and after the application of tDCS. The stimulus presentation rate was selected individually for each participant, but was then maintained constant between sessions. The instructions for the PASAT were similar to those of the traditional version of the task, which principally involved instructing participants to *add* the number they just heard to the number they heard before. In contrast, the instructions for the PASST involved instructing participants to *subtract* the number they just heard from the number they heard before. Participants were allowed a short break between each task (approximately 30 s), which each lasted approximately 10 min. Each answer was written down by the experimenter for subsequent verification, and correct answers were checked against a printed score sheet. No score was given if a participant gave an incorrect answer or failed to respond.

### Data analysis

The results for both experiments were analysed primarily in terms of the numbers of correct responses (accuracy scores), and the mean and variability (standard deviation) of participants’ verbal response times, before (session one) and after (session two) the application of sham, cathodal and anodal cerebellar stimulation. Accuracy scores were also normalized to negate an effect of stimulus presentation rate. This was achieved by dividing the total number of correct responses by the rate at which participants preformed each task. Two participants from each group were excluded from analyses of response times because they failed to complete both experiments before and after stimulation. Only responses associated with correct answers were analysed. Reasons for excluding data included: incorrect responses, missed responses, double responses (i.e. a response preceded by lip movement/breath of air) or inaudible/undetected responses). In experiment two, responses that exceeded ±2SD of the mean were also excluded to avoid analysing prolonged answers. The total amount of data excluded from data analyses was no more than 36% (32% incorrect, 4% missed/double responses) for any one participant in experiment one, and 8% in experiment two.

## Results and discussion, experiment one

### Stimulus presentation rate

An analysis was first performed to assess whether pre-stimulation performance on each task was influenced by the participant-specific stimulus presentation rates that could have varied between groups. Given the unequal cognitive demands of performing the two paced tasks, participants’ verbal responses were slower during subtraction (2.70 s) than during addition (2.34 s) versions of the task as revealed by a main effect of Task (*F*_1,63_ = 64.46, *P* < 0.001). However, stimulus presentation rate did not differ significantly between the three groups (sham, anodal and cathodal, 2.56, 2.50 and 2.49 s, respectively, *F*_2,63_ = 0.23 *P* = 0.79), as confirmed with pair-wise *t*-tests adjusted for multiple comparisons. Furthermore, there was no Group × Task interaction *F*_2,63_ = 0.60, *P* = 0.55).

### Subjectivity ratings

Subjective difficulty from 15 participants ratings were compared between all tasks. This analysis revealed a main effect of Task (*F*_4,56_ = 109.73, *P* < 0.001), such that the subtraction task was rated significantly more difficult to perform than the addition task (7.53 vs. 5.60). Both arithmetic tasks were rated more difficult to perform than the verb generation (3.47), noun reading (1.33) and verb reading (1.33) tasks, the latter two of which were not significantly different from one another.

### Accuracy scores

**Figure 1:**
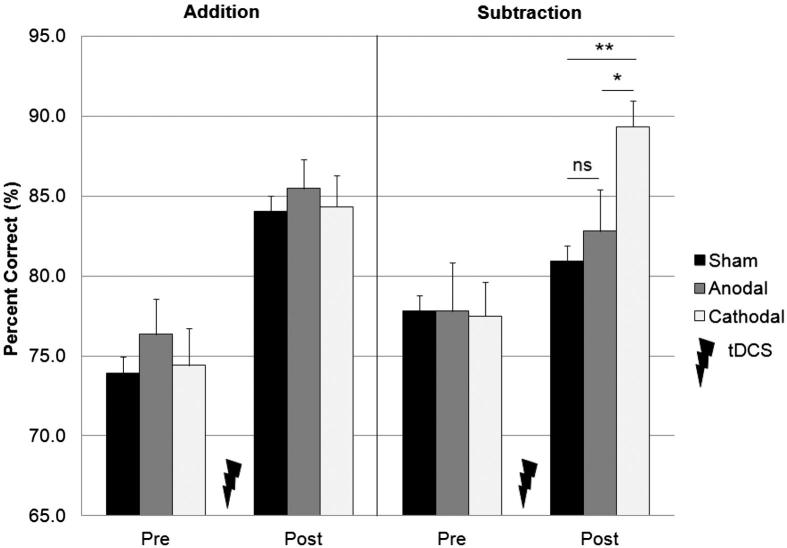
Mean accuracy (+1 SE of the mean, group *n* = 20) in the addition (PASAT) and subtraction (PASST) tasks, before and after cerebellar tDCS. The number of correct answers that participants obtained on the subtraction task, but not the addition task, was significantly greater after cathodal, than after anodal or sham stimulation. Asterisks indicate significant differences (*P* < 0.05) as revealed with post hoc *t*-tests.

**Figure 2:**
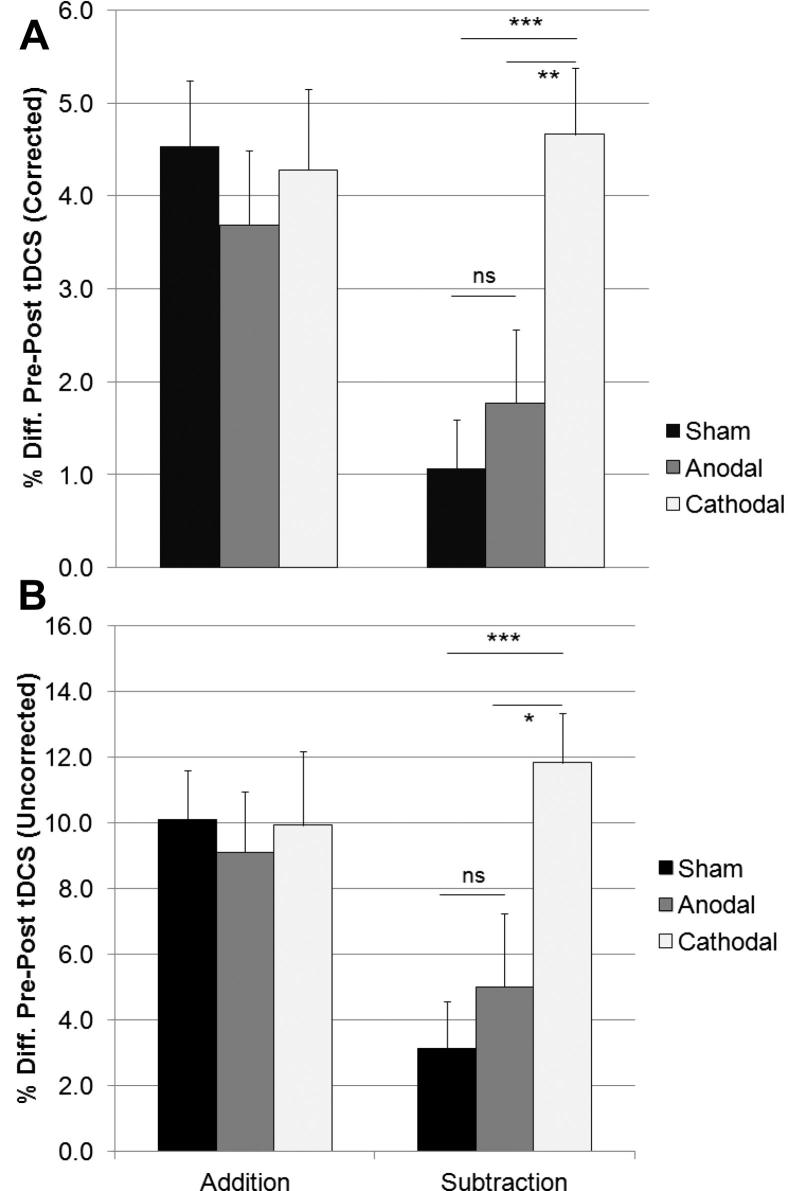
Increase in accuracy (mean + 1 SEM, *n* = 20) from session one (pre-stimulation) to session two (post-stimulation), in the addition (PASAT) and subtraction (PASST) tasks (A). Increases in percentage accuracy, normalized by the stimulus presentation rates (B). Individual stimulus rates were selected during a practice session but held constant across sessions one and two. This figure emphasizes the gain in accuracy that participants’ experienced between stimulation sessions on the subtraction task, but not the addition task after cathodal stimulation only, and shows how the result is unaffected by the specific rate at which participants performed each task. Asterisks indicate significant differences (*P* < 0.05) as revealed with post hoc *t*-tests.

Fig. 1 summarizes participants’ accuracy (expressed as percent correct) in the addition and subtraction tasks, before (session one) and after (session two) the application of sham, cathodal or anodal stimulation. A 2 × 3 (Session × Group) ANOVA demonstrated a main effect of Session (*F*_1,63_ = 109.24, *P* < 0.001), such that the number of correct answers increased on session two (84.47%) compared with session one (76.30%), presumably due to practice. Of particular interest was the Task × Session × Group interaction that was significant (*F*_2,63_ = 3.36, *P* < 0.05), and was due to the increased number of correct answers on the subtraction task, but not the addition task, seen after cathodal stimulation (77.50 vs. 89.32%). The increase was smaller for the anodal (77.80 vs. 82.80%) or sham (77.81 vs. 80.91%), stimulation groups (Fig. 2A). Furthermore, this pattern was still present when accuracy data for each task were normalized by each participants’ stimulus presentation rate (see Fig. 2B).

### Verbal response times

**Figure 3:**
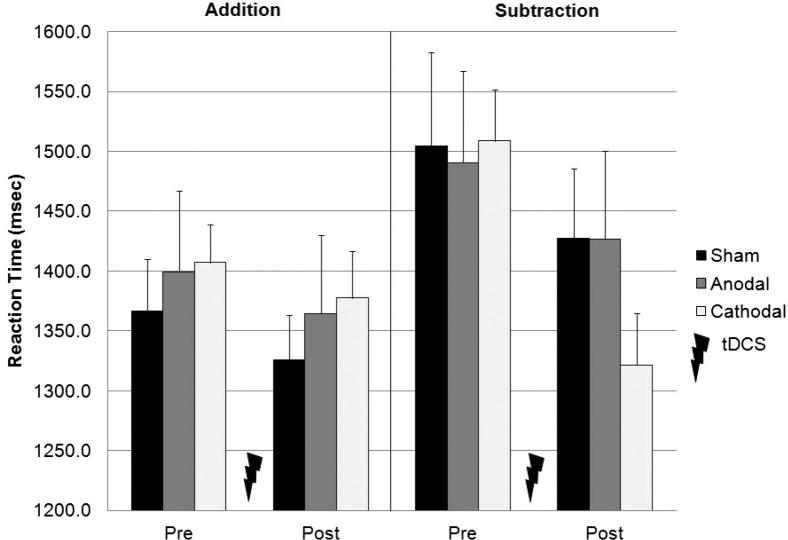
Mean response latency (+1 SE of the mean, group *n* = 20) in the addition (PASAT) and subtraction (PASST) tasks, before and after cerebellar tDCS. There was a trend for mean response times on the subtraction task, but not the addition task, to decrease more after cathodal, than after anodal or sham stimulation. The change in response speed between pre- and post-tDCS sessions for each task is more clearly shown in Fig. 4.

**Figure 4:**
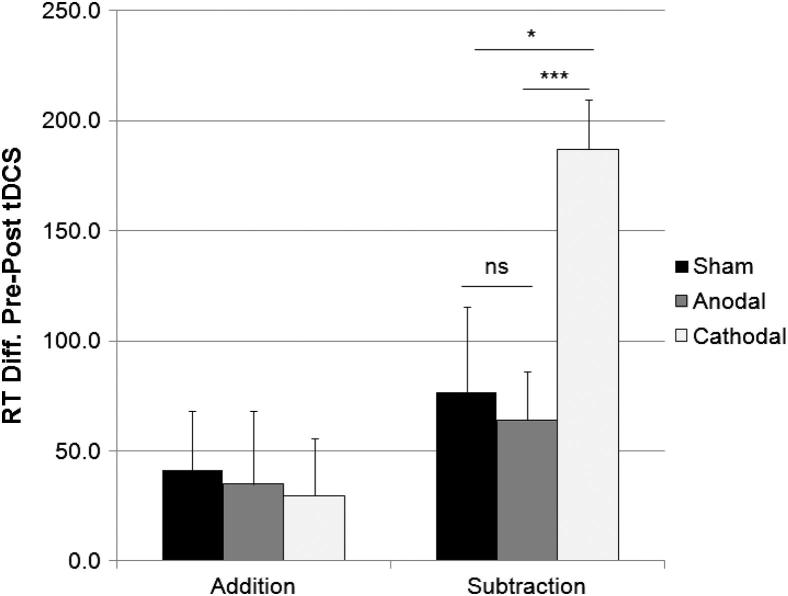
Improvement in response speed (mean + 1 SEM, *n* = 20) from session one (pre-stimulation) to session two (post-stimulation), in the addition (PASAT) and subtraction (PASST) tasks. Participants performed calculations more quickly after cathodal, than after anodal or sham stimulation on the subtraction task, but not the addition task. Asterisks indicate significant differences (*P* < 0.05) as revealed with post hoc *t*-tests.

An analysis of participants’ mean verbal response times provides a measure of how quickly they produced correct answers (Fig. 3). Mean response times were faster during addition than during subtraction (1372 vs. 1447 msec; *F*_1,57_ = 11.70, *P* < 0.001), and decreased after stimulation (1446 vs. 1374 msec; *F*_1,57_ = 36.43, *P* < 0.001). Complementing the results from the analysis of participants’ accuracy scores, the Task × Session × Group interaction was close to significant (*F*_1,57_ = 2.65, *P* = 0.08), and were due to participants’ response times on the subtraction task decreasing more after cathodal stimulation (1509 vs. 1322 msec), than after anodal (1491 vs. 1427 msec) or sham (1504 vs. 1427 msec) stimulation (see Fig. 4). The reduction in response times in session two was equal across the three stimulation groups for the addition task.

### Response time variability

**Figure 5:**
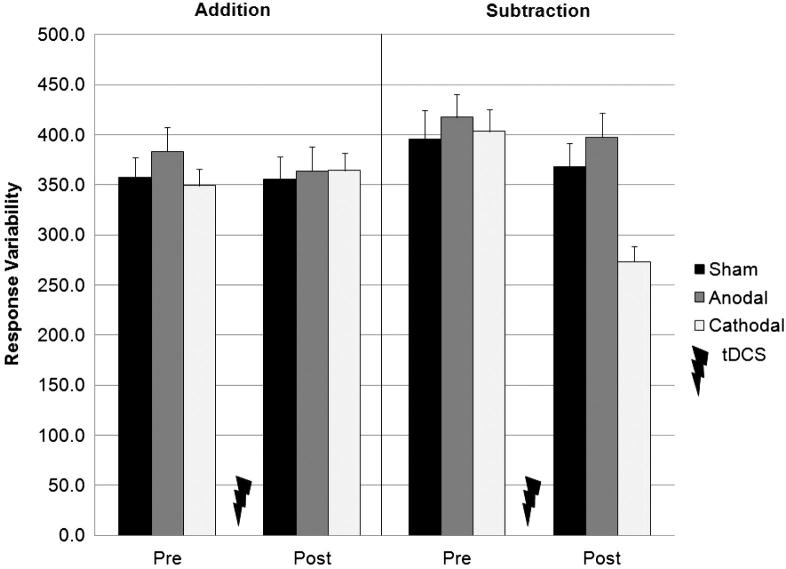
Mean response latency variability (mean SD + 1 SE of the group mean, *n* = 20) in the addition (PASAT) and subtraction (PASST) tasks, before and after cerebellar tDCS. The variability of participants’ response times on the subtraction task, but not the addition task, decrease significantly more after cathodal, than after anodal or sham stimulation. The change in response latency variability between pre- and post-tDCS for each task is more clearly shown in Fig. 6.

**Figure 6:**
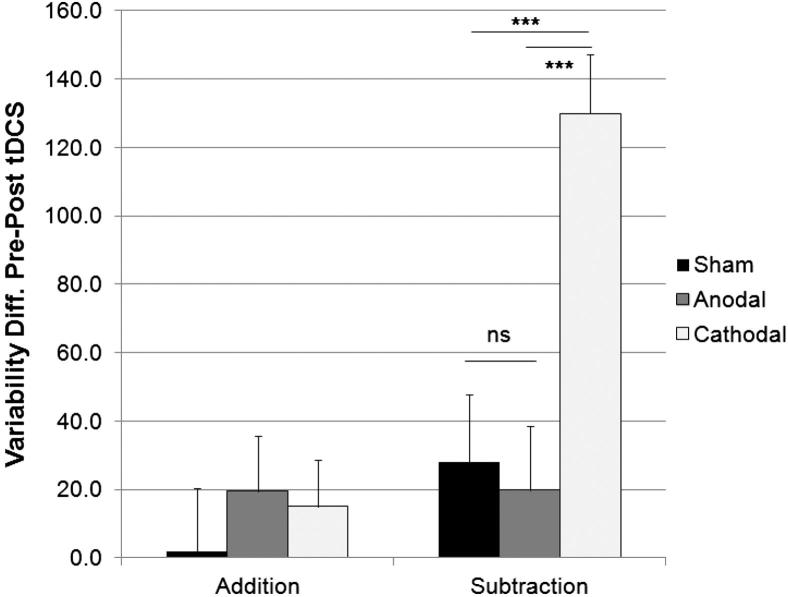
Reduction in response latency variability (mean SD + 1 SEM, *n* = 20) from session one (pre-stimulation) to session two (post-stimulation), in the addition (PASAT) and subtraction (PASST) tasks. The speed that participants performed calculations was more consistent after cathodal, than after anodal or sham stimulation on the subtraction task, but not the addition task. Asterisks indicate significant differences (*P* < 0.05) as revealed with post hoc *t*-tests.

An analysis of the variability (standard deviation) of participants’ verbal response times provides a measure of how consistently they produced correct answers on each task. These values (Fig. 5) shows significant decrease in response variability between sessions one (386 msec) and two (354 msec; *F*_1,57_ = 16.86, *P* < 0.001). This pattern of results was significantly different for each task, (*F*_1,57_ = 17.46, *P* < 0.001), and each group, (*F*_2,57_ = 3.20, *P* < 0.05) as revealed by respective Session × Task, and Session × Group interactions. As expected, the Task × Session × Group interaction was also significant (*F*_2,57_ = 11.16, *P* < 0.001), and was due to the variability of participants’ responses on the subtraction task decreasing more after cathodal (403 vs. 273 msec), but less so after anodal (418 vs. 398 msec) or sham (396 vs. 368 msec), stimulation (see Fig. 6). As above, the reduction in response time variability for the addition task were equal across the three stimulation groups.

In summary, the results from experiment one demonstrate that cathodal DC stimulation applied over the right cerebellar hemisphere selectively enhanced performance on a subtraction version of the paced serial addition task. Changes in performance on this task after cathodal tDCS included a significant improvement in the number of correct scores between sessions, and calculations that were performed faster and with less variable latencies. The subtraction task was rated as significantly more difficult to perform than the addition task, implying that stimulation of the cerebellum affects performance on tasks that are more challenging to perform.

## Experiment two: working memory for language processing

The role of the cerebellum in verb generation has been debated since the work by [15], showing impairment in a single case study after right cerebellar stroke. Brain imaging studies have since shown that the right cerebellar hemisphere is active when participants are required to generate appropriate verbs in response to target nouns [17]. However, patients with cerebellar damage can perform the same task as well as healthy controls [25,45]. Can performance on this task be perturbed by stimulating the right cerebellar hemisphere? To test this question, the same three groups of participants each performed a noun reading, a verb generation and a verb reading task, before (session one) and after (session two) the application of anodal, cathodal or sham stimulation over the right cerebellum.

### Materials

The stimuli consisted of one list of 40 concrete nouns related to manipulable tools/objects, and a corresponding list of 40 concrete verbs related to tool/object manipulation. Half the words in this list were presented in session one, and the other half in session two. The final lists were generated on the basis of verb generation data from an independent group of subjects (*N* = 35). Only noun–verb pairs generated by more than half of the pilot group (median = 79%) were selected for inclusion in the experiment. Nouns were avoided if they generated the same verb (e.g. dinner-eat, banana-eat) or produced verbs that were passive (e.g. bed-sleep) or did not refer to physical acts performed by humans (e.g., oven-bake). Each task consisted of 6 blocks of 20 trials each. The same words were used for blocks 1–5 (repeated words), yet presented in a different random. In block 6, a new set of words was presented (novel words). The word lists in each session were different, and counterbalanced across participants.

### Procedure

Experiment two was performed approximately 1 min after experiment one was completed. The experiment consisted of a noun reading, a verb generation and a verb reading task, before (session one) and after (session two) the application of cerebellar stimulation. At the start of each block, the word READY appeared in the centre of a computer screen. On each trial, a word was selected from the current word list at random (without replacement) and presented centrally on the screen. The word remained on the screen until the microphone recorded a response. In the noun and verb reading tasks, participants were instructed beforehand to read the presented word aloud as soon as it appeared on the screen. In the verb generation task, participants were instructed beforehand to say an appropriate verb (e.g. cut) in response to the presented noun (e.g. scissors). An appropriate verb was defined as one that describes what the presented noun might do, or what it might be used for. Participants were instructed to produce words as quickly as possible, and were not informed that they might be repeated. All participants were given a practice set of 5 items at the beginning of each task that did not appear in the experiment. Participants were allowed a short break between tasks (approximately 10 s), which each lasted approximately 5 min. The accuracy of each word spoken during the noun and verb reading tasks were checked by the experimenter against those presented on the computer screen. Verbs produced during the verb generation task were written down by the experimenter for subsequent verification. If a participant made an inappropriate response or no response the error was noted, and the participant was told to continue. Response times were calculated off-line.

## Results and discussion, experiment two

### Response accuracy

Participants made very few errors in experiment two (<8% in any session or group), and so these data were not analysed in any detail.

### Verbal response times

**Figure 7:**
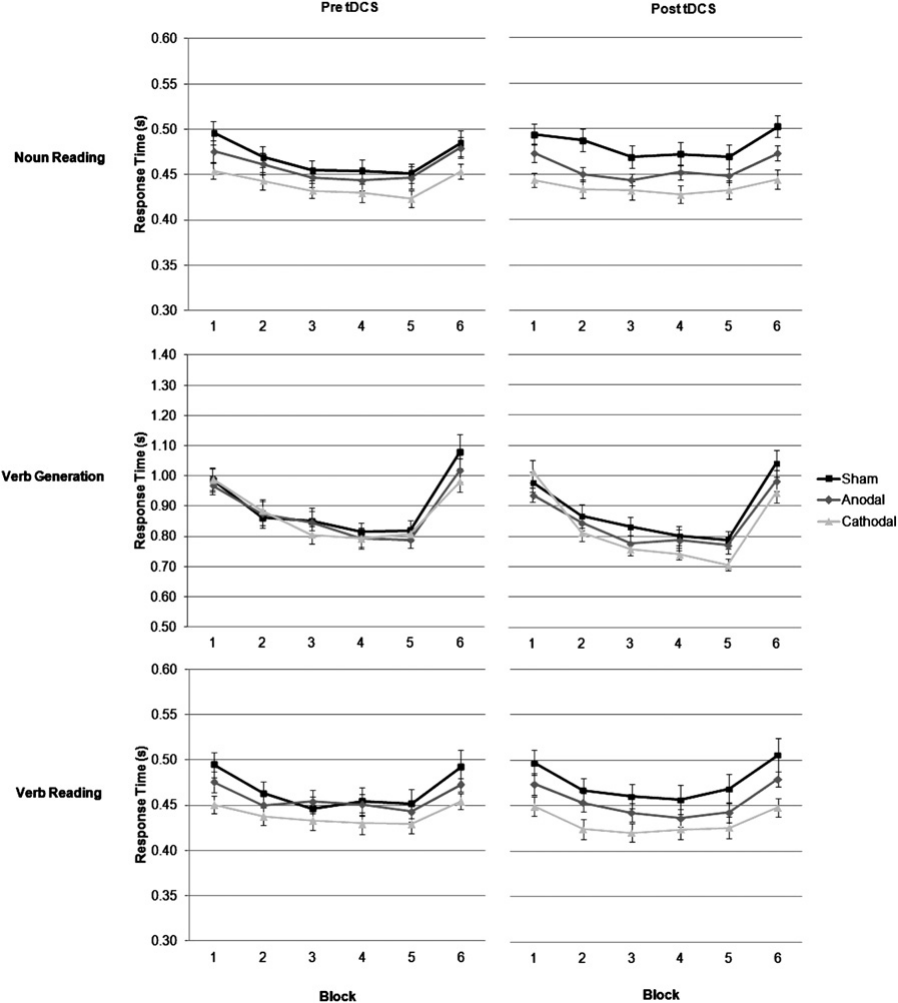
Change in mean response latency (mean + 1 SEM, *n* = 20) across the 6 blocks of trials in the word reading and verb generating tasks. The word lists were repeated across blocks 1–5, and new word lists introduced in Block 6. Different word lists were used between sessions one and two. The response latency decreased more between blocks of repeated words during the verb generation task after cathodal, than after anodal or sham stimulation. This difference in performance between lists of repeated words is shown more clearly in Fig. 9.

Fig. 7 shows participants’ mean verbal response times for the three Groups (sham vs. anodal vs. cathodal) across trial blocks (1–6), Task (noun reading [upper row] vs. verb generation [middle row] vs. verb reading [lower row]), and Session (before [left column] vs. after [right column]). A Group × Block × Task × Session ANOVA revealed a main effect of Task (*F*_2,114_ = 1086.10, *P* < 0.001), such that response times were slower during verb generation (0.87 s), than during noun (0.46 s) and verb (0.45 s) reading tasks. Response latencies improved within the first five blocks of repeated words (*F*_5,285_ = 146.47, *P* < 0.001), an effect of priming, then increased in block 6 where new words were introduced. This pattern of priming was different for each task, as revealed by a significant Task × Block interaction, (*F*_10,570_ = 74.72, *P* < 0.001), and for each session, as revealed by a significant Task × Session interaction, (*F*_2,114_ = 6.71, *P* < 0.01). A main effect of Session was close to significance, (*F*_1,57_ = 3.70, *P* = 0.06), such that response times decreased slightly between sessions one and two (0.62 vs. 0.61 s), together with a main effect of Group, (*F*_2,57_ = 2.45, *P* = 0.09), such that response times got progressively slower between sham (0.61 s), anodal (0.59 s) and cathodal (0.58 s) stimulation. The Group × Block × Task interaction was significant (*F*_20,570_ = 1.83, *P* < 0.05), but there was no Session × Task × Group interaction, or other significant effects.

### Response variability

**Figure 8:**
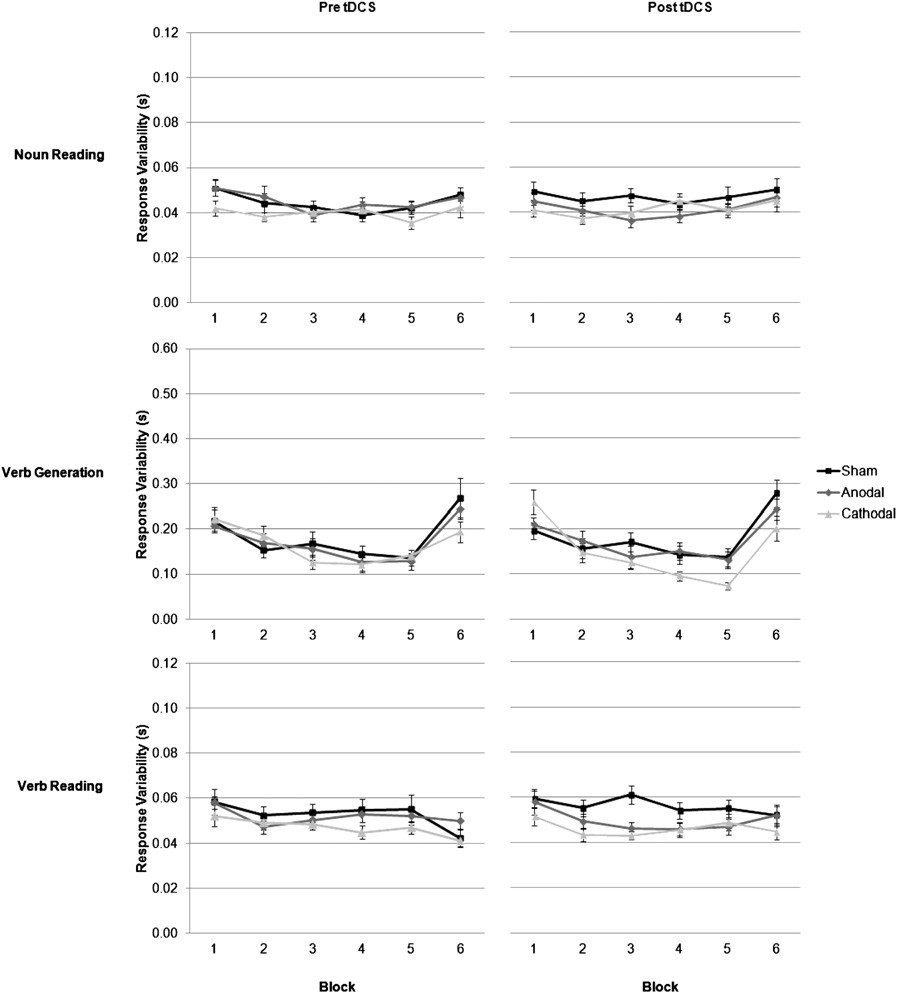
Change in mean response latency variability (mean SD + 1 SEM, *n* = 20) across the 6 blocks of trials in the word reading and verb generating tasks. The word lists were repeated across blocks 1–5, and new word lists introduced in Block 6. Different word lists were used between sessions one and two. The response latency variability decreased more between blocks of repeated words during the verb generation task after cathodal, than anodal or sham stimulation. This aspect of performance is shown more clearly in Fig. 10.

The variability of participants’ mean verbal response times across Block, Task and Session and averaged by Group are plotted in Fig. 8, and shows how response variability was influenced by Task (*F*_2,114_ = 325.93, *P* < 0.001), such that response latencies were more variable during verb generation (0.17 s) than during noun (0.04 s) and verb (0.05 s) reading tasks. Variability reduced within each task, as reflected in a main effect of Block (*F*_5,285_ = 45.55, *P* < 0.001), where response variability decreased significantly across the 5 blocks of repeated words, then increased in block 6, when new word lists were introduced. Again, this pattern of priming was different for each task, as response variability was reduced most during repeated verb generation than in the two reading tasks, as revealed by significant Task × Block interaction, (*F*_10,570_ = 36.18, *P* < 0.001). Response variability between blocks also varied across groups as revealed by a significant Group × Block interaction, (*F*_10,285_ = 2.30, *P* < 0.05), and varied as a function of task as revealed by a significant Group × Task × Block interaction, (*F*_20,570_ = 2.57, *P* < 0.001). However, there was no effect of Session or a Session × Task × Group interaction, or other significant effects.

### Learning

**Figure 9:**
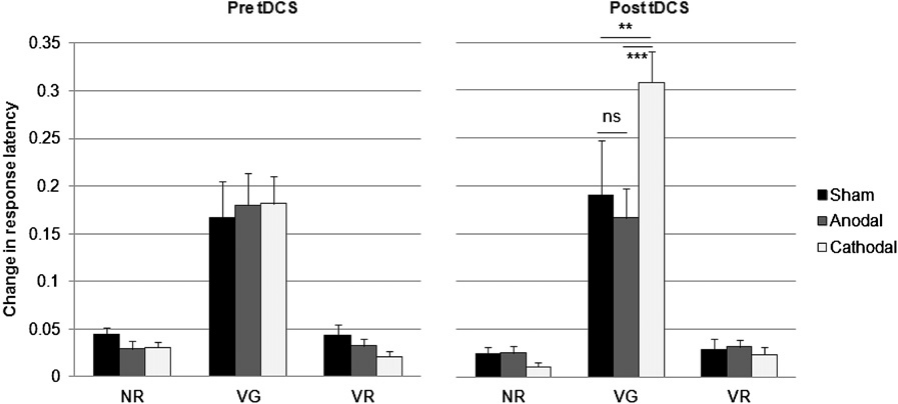
Increase in response speed (mean + 1 SEM, *n* = 20) between blocks 1–5 (mean total learning) for the noun reading (NR), verb reading (VR) and verb generating (VG) tasks. As words were repeated across blocks 1–5, participants generated responses more quickly after cathodal, than after anodal or sham stimulation during verb generation, but did not change for the two reading tasks. Asterisks indicate significant differences (*P* < 0.05) as revealed with post hoc *t*-tests.

In addition to comparing differences between absolute means, the total amount of learning within each task, before and after stimulation, was quantified by comparing participants’ mean response times between the first (Block 1) and last (Block 5) set of repeated words for each group (see Fig. 9). A Task × Session × Group ANOVA revealed a main effect of Task (*F*_2,114_ = 128.88, *P* < 0.001), such that the difference in response times between blocks 1–5 was greater for verb generation (0.20 s), than for noun (0.03 s) and verb (0.03 s) reading tasks. The total amount of learning for each task was different for each session, as revealed by a significant Task × Session interaction (*F*_2,56_ = 7.19, *P* < 0.001), and for each group, as revealed by a significant Task × Group interaction, (*F*_4,114_ = 3.20, *P* = 0.05). The Session × Group interaction was also significant (*F*_2,57_ = 4.39, *P* = 0.05). Of interest, the Session × Task × Group interaction was significant (*F*_4,114_ = 4.50, *P* = 0.01), such that the total amount of learning on the verb generation task increased more after cathodal (0.18 vs. 0.31 s), than after anodal (0.18 vs. 0.17 s) or sham (0.17 vs. 0.19 s) stimulation. There were no other significant effects.

### Change in variability

**Figure 10:**
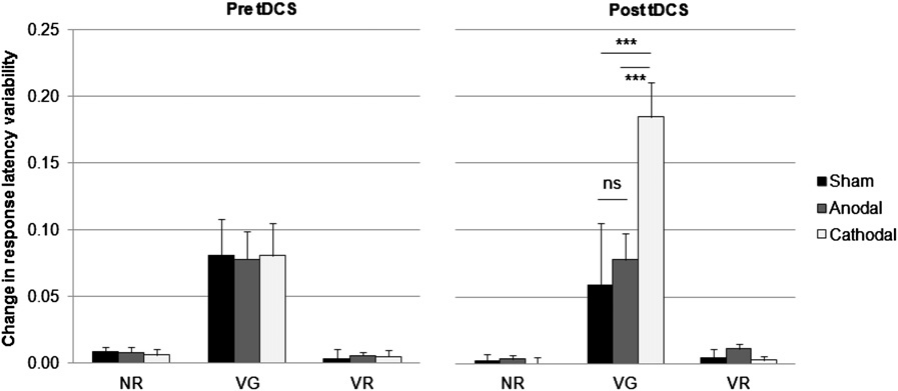
Reduction in response latency variability (mean SD + 1 SEM, *n* = 20) between blocks 1–5 (total learning variability) for the noun reading (NR), verb reading (VR) and verb generating (VG) tasks. The latency with which participants generated responses was more consistent after cathodal, than after anodal or sham stimulation during verb generation, but not after the two reading tasks. Asterisks indicate significant differences (*P* < 0.05) as revealed with post hoc *t*-tests.

The difference in mean response latency variability between blocks 1–5 (total learning variability) within each Task, Session and averaged by Group are plotted in Fig. 10, and shows how response variability between the first and last block was influenced by Task (*F*_2,114_ = 64.32, *P* < 0.001), such that the change in response variability between blocks 1–5 was greater for verb generation (0.09 s), than for noun (0.005 s) and verb (0.006 s) reading tasks. A main effect of Group was also significant, (*F*_2,57_ = 3.21, *P* < 0.05), such that total response latency variability was reduced more after cathodal (0.05 s), than after anodal (0.03 s) or sham (0.03 s) stimulation. The change in variability for each group was also different for each session, as revealed by a significant Session × Group interaction, (*F*_2,57_ = 4.09, *P* < 0.05), and for each task, as revealed by a significant Task × Group interaction, (*F*_4,114_ = 3.51, *P* < 0.01). The Session × Task × Group interaction was also significant (*F*_4,114_ = 5.19 *P* < 0.001), such that the change in response variability between blocks on the verb generation task was greater after cathodal (0.08 vs. 0.19 s), than after anodal (0.08 vs. 0.08 s) or sham (0.08 vs. 0.06 s) stimulation. There were no other significant effects.

In summary, the results from experiment two demonstrate differences in the mean and variability of participants’ verbal response times both within and between each of the three language tasks. All three groups showed significant improvement in performance over repeated word lists, but more importantly there was a selective facilitatory effect of cathodal DC stimulation on performance during the verb generation task. Changes in performance within this task after cathodal tDCS included the generation of action-related verbs that were performed faster and with less variable latencies.

## General discussion

A role for the cerebellum in cognition is controversial, but it is a view that is becoming increasingly popular [49,51], despite criticism that results are sometimes inconsistent or confounded by motor responses [57]. The present study was set up to investigate whether modulating the activity of the cerebellum using DC stimulation could influence performance in two cognitive tasks that have previously been shown to activate the cerebellum in an MR scanner. The arithmetic tasks in experiment one involved different amounts of working memory and attention but similar motor responses. We demonstrated a facilitatory effect of cathodal tDCS (relative to anodal and sham stimulation) on participants’ accuracy scores, and on the mean and variability of their response latencies, such that verbal responses were more accurate, faster and less variable after stimulation. Interestingly, these facilitatory effects were only seen in the subtraction version of the arithmetic task, which was more difficult to perform than the additive PASAT task. Comparable effects were also seen in experiment two, where cathodal stimulation of the cerebellum facilitated performance of verb generation, relative to noun and verb reading tasks. The results for each experiment are discussed in a broader context below.

This study is the first to publish data relating to performance on a subtraction version of the PASAT. The justification for including this version was to have a task that was motorically similar to the PASAT, but required more effort to perform. We hypothesised that we would then see a differential effect in the two tasks, if the cerebellum was contributing both to cognitive and to motor performance. The data from both experiments did indeed show that it is possible to affect performance using DC stimulation of the cerebellum. More specifically, cathodal stimulation of the right cerebellar hemisphere was able to facilitate behavioural measures of performance during an arithmetic cognitive task, and a verb generation task, whereas the effect of anodal stimulation was no different from sham stimulation.

The motor requirements of the PASAT and PASST tasks are comparable, but the mental operations required to perform subtraction versus addition are very different. For example, order effects are relevant in subtraction (4 minus 3 is not the same as 3 minus 4), whereas they are irrelevant in addition. This is one reason why subtraction is considered more difficult to perform than addition [18]. By individualising the stimulus presentation rates for each task, participants were able to perform the PASST at a comparable level of accuracy to the PASAT, albeit a little slower and with greater variability in their response latencies. Thus baseline accuracy in the PASAT and PASST tasks was comparable (Fig. 1), and performance in both improved in session two, reflecting increased practice. However, after cathodal stimulation, participants were able to perform the more challenging subtraction task at a higher level of accuracy, faster and more consistently than any of the three groups performing the easier addition task. This result cannot be explained by any change in cerebellar contribution to motor performance. It suggests instead that effects of tDCS on cognitive behaviour are likely task- or load-specific.

Complimenting the results from experiment one, DC stimulation of the cerebellum also differentially affected performance on the language tasks investigated in experiment two. Namely, cathodal stimulation facilitated performance on the verb generation task, relative to performance that was unchanged by stimulation of any kind on the relatively easier noun and verb reading tasks. This was evidenced by response latencies that were faster and less variable (priming effects) between repeated exposure to the noun lists used. Previously, tDCS over the cerebellum has been to shown to influence motor adaptation [20], whereas cerebellar disruption using TMS has been shown with lengthened RTs during a verbal working memory task [9]. Our data are also congruent with those from fMRI studies in which cerebellar activity has been observed in healthy subjects during verbal working memory tasks [5,8,10], and in patients with cerebellar lesions where verbal working memory has been shown to be impaired [14,44]. Coupling MRI with cognitive performance in patients with cerebellar degeneration (SCA-6) has also revealed how verbal working memory is related to grey matter density in superior and inferior parts of the cerebellum [7]. These data are consistent with a proposed cerebrocerebellar network supporting verbal working memory [10].

Further support for a role of the cerebellum in language is grounded in the concept of embodied cognition, which asserts that the motor system may participate in the production of words related to actions, as it is also engaged during the production of those same actions (reviewed in Ref. [41]). For example, activity is observed in premotor and primary motor cortices for silent reading of words referring to face-, arm- or leg-related actions [23], Similarly, activity in the cerebellum is observed when subjects are instructed to imagine articulating words [1], and when generating verbs silently [17]. It would appear that the cerebellum is recruited not just for coordinating the execution of movements, but also for coordinating higher cognitive functions associated with producing speech.

Our data suggest that the cerebellum is capable of influencing behaviour when cognitive tasks make high demands on working memory and attention resources. Individual participant’s task difficulty or effort is not often assessed in experiments, whether they investigate motoric or cognitive skills. Thus, the extent to which a participant engages cognitive resources related to effort is often unknown, and presumably varies considerably between tasks and participants. Our design ensured that each participant performed both tasks in experiment one at similar difficulty; had participants performed close to ceiling on session one, a facilitating effect of tDCS on sessions two would not be observed.

Indeed, the extent to which participants engage in an experiment is something that should be considered when running neurostimulation studies, as the brain is presumably less engaged, and therefore less activated, when subjects find a task easy. Brain imaging studies reveal how activity in a cognitive network comprising the parietal and dorsolateral prefrontal cortex is positively correlated with measures of increasing task complexity such as reasoning and problem-solving during cognitive tasks [31]. The results from the current study suggest that tDCS is more effective when participants have to fully engage with a difficult task, or when they find it difficult to perform.

Our data strengthen the view that the cerebellum is capable of influencing cognition under certain circumstances. We speculate that the cerebellum is capable of releasing cognitive resources in working memory regions of cortex by dis-inhibition of the dorsolateral prefrontal cortex: cathodal cerebellar tDCS would hyperpolarize cerebellar cortex, reducing the Purkinje cell outputs which normally exert an inhibitory tone on the cerebral cortex [19]. Indeed, the dorsolateral prefrontal cortex is engaged in many cognitive tasks and is known to be critical for working memory and attention. In the monkey it has reciprocal connections with lateral portions of the cerebellar hemisphere, as identified with cell-tracking methods (reviewed in Ref. [56]), and is thought to be similarly connected in humans (reviewed in Ref. [43]). Studies that have previously demonstrated facilitatory effects of tDCS on tests of mathematics and language have done so using anodal stimulation elsewhere in the brain [6]. The enhancement of mathematics and language performance observed in this study using cathodal stimulation over the cerebellum may at first appear contradictory to these previous findings. However, it should be noted that connections between the cerebellum and motor and prefrontal regions of cortex are via the inhibitory Purkinje cells of the cerebellar cortex. Thus, cathodal stimulation is expected to inhibit this inhibitory output from the cerebellum to the prefrontal cortex, making the latter region (typically associated with working memory functions) more active, which can partly explain the facilitatory effects of tDCS on cognition observed in the present study.

An important point to consider when interpreting the current study (and other investigations of cerebellar non-motor functions), is the view that the cerebellum may provide functional support for many neural operations, but may not itself participate in their computations [4]. In other words, activity in the cerebellum is not related to cognition *per se*, but that cognitive deficits after cerebellar damage are thought to reflect effects elsewhere in the cerebral cortex induced by the loss of cerebellar input. According to this supposition, the cerebellum plays a role in cognition by influencing excitability and thus processing in prefrontal regions of cortex, and in turn is selectively able to facilitate performance when cognitive tasks become difficult to perform. Thus, the cerebellum may be amplifying the prefrontal areas to facilitate their roles in cognitive operations.

Evidence from lesion studies has shown that the cerebellum is involved in the process by which novel motor tasks can, after some practice, be performed automatically and skilfully (reviewed in [12]). Because the cerebellum is connected to regions of the brain that perform motor, mental and sensory tasks, it might automatize not only motor, but also cognitive operations in the brain that require mental and sensory information [29,43]. However, it is unlikely that our results from experiment one are due to automatization of arithmetic aspects of the tasks, since the specific calculations participants’ performed during each arithmetic task in the PASAT and PASST tasks are unpredictable, and different between sessions. Nonetheless, a role for the cerebellum in the automatization of cognitive skill is congruent with our results from experiment two, where response latencies were faster and less variable (priming effects) within the verb generation task after cathodal stimulation.

It is perhaps surprising that the application of tDCS over the cerebellum did not influence performance on the PASAT, which is widely acknowledged to be a difficult task to perform, which recruits brain structures like the prefrontal regions of cortex, as well as the cerebellum [24]. Our results do not dispute the role of the cerebellum during the PASAT, but may mean that the effect of modulating this structure with tDCS may not lead to detectable changes in performance, if there are enough cognitive resources available for executing the task correctly. It is also clear that anodal tDCS had no effect, and the changes in performance seen in the anodal group were identical to those seen in the sham group. This implies that any changes in cerebellar cortical excitation did not result in functional inhibition of the prefrontal cortex. Other research also suggests a strong asymmetry in the effects of cerebellocerebral inhibition [34].

In conclusion, cathodal tDCS applied over the right cerebellum facilitated performance on an arithmetic and verb generating task that both required a high level of cognitive load compared with arithmetic and reading tasks that require less effort, in which tDCS has no added benefit. We suggest that modulation of the cerebellar cortex is capable of enhancing performance when cognitive tasks become difficult by releasing additional resources from prefrontal regions of cortex.
